# Evaluation of different scoring systems in the prediction of complications, morbidity, and mortality after laparoscopic radical gastrectomy

**DOI:** 10.1186/s12957-023-03282-5

**Published:** 2023-12-18

**Authors:** Haoyu He, Yubiao Liu, Xin Liu, Zhaoxiong Zhang, Daohan Wang, Weihua Fu

**Affiliations:** 1 Department of General Surgery, Tianjin Medical University General Hospital, 154 Anshan Road, Tianjin, 300052 People’s Republic of China; 2 Department of Anorectal Surgery, Liaocheng People’s Hospital, Liaocheng, Shandong China

**Keywords:** Laparoscopic radical gastrectomy, Surgical scoring system, Postoperative complications, Morbidity and mortality

## Abstract

**Background:**

This retrospective study aimed to assess the suitability of POSSUM and its modified versions, E-PASS and its modified score, SRS, and SORT scores for predicting postoperative complications and mortality in patients undergoing laparoscopic radical gastrectomy for gastric cancer.

**Materials and methods:**

Data analysis was performed on 349 patients who underwent laparoscopic radical gastrectomy at Tianjin Medical University General Hospital between January 2016 and December 2021. The discriminative ability of the scoring systems was evaluated using the area under the receiver operating characteristic curve (AUC). The primary endpoint focused on the prediction of postoperative complications, while the secondary endpoint assessed the prediction of postoperative mortality.

**Results:**

Among the scoring systems evaluated, the modified E-PASS (mE-PASS) score exhibited the highest AUC (0.846) and demonstrated the highest sensitivity (81%) and specificity (79%) for predicting postoperative complications. All other scores, except for POSSUM, showed moderate discriminative ability in predicting complications. In terms of predicting postoperative mortality, the E-PASS score had the highest AUC (0.978), while the mE-PASS score displayed the highest sensitivity (76%) and specificity (90%). Notably, both E-PASS and mE-PASS scores exhibited excellent discriminative ability.

**Conclusions:**

The P-POSSUM, O-POSSUM, E-PASS, mE-PASS, SRS, and SORT scoring systems are useful tools for predicting postoperative outcomes in laparoscopic radical gastrectomy. Among them, the mE-PASS score demonstrated the best predictive power. However, the POSSUM system could only be applicable to predict postoperative mortality.

## Introduction

Gastric cancer (GC) continues to be one of the most lethal cancers globally, ranking fifth in terms of incidence and fourth in terms of mortality [1]. Laparoscopic gastrectomy has emerged as a widely employed approach for GC treatment, offering a more promising outlook and well-defined hierarchical structure compared to traditional laparotomy [2]. However, the occurrence of postoperative complications remains a significant challenge that impacts patient prognosis.

The occurrence of postoperative complications in GC is influenced by multiple factors. In the context of conventional open surgery, various surgical scoring systems have been developed to predict the morbidity and mortality associated with these complications. Initially, the Physiological and Operative Severity Score for the enUmeration of Mortality and Morbidity (POSSUM) score was introduced to assess the risk of postoperative complications and evaluate surgical quality. The POSSUM score comprised two components: the physiology score (PS) and the operative score (OS). The physiology score incorporates 12 indicators, while the operative severity score encompasses 6 indicators. Each indicator was categorized into four grades and derived using a binary logistic regression Eq. [3]. However, it was observed that POSSUM tends to overestimate postoperative mortality [4]. Whiteley et al. [5] addressed this issue by making adjustments to the POSSUM equation, resulting in the development of the P-POSSUM score, which exhibited enhanced accuracy in predicting postoperative mortality. Additionally, due to the relatively high rates of complications and mortality associated with esophageal and gastric surgeries, O-POSSUM was specifically developed to improve the predictive capabilities of POSSUM for these procedures [6]. Recognizing the significance of patients’ physiological status and their response to surgical trauma, Haga et al. [7] introduced the Estimation of Physiologic Ability and Surgical Stress (E-PASS) scoring system. This predictive tool assesses postoperative risk by evaluating the patient’s physiological reserve and surgical stress. The E-PASS scoring system comprised three components: the preoperative risk score (PRS), which primarily reflects the patient’s physiological status; the surgical stress score (SSS), which primarily evaluates the patient’s ability to cope with the stress of surgery; and the comprehensive risk score (CRS). A modified version of E-PASS, known as mE-PASS, incorporates various surgical procedures to calculate a median score for the proposed surgical method, thereby obtaining the surgical stress score specific to that surgery. Building upon E-PASS, mE-PASS was subsequently developed to simplify data entry while maintaining its applicability, and its national validation has been conducted [8].

In addition, Sutton et al. [9] developed a simplified surgical risk scoring system. The surgical risk score (SRS) comprised three preoperative variables: surgical urgency grading, as defined by the Confidential Enquiry into Perioperative Deaths (CEPOD) grade; surgical size grading, as defined by the British United Provident Association (BUPA); and ASA grading. The SRS score for each patient was determined preoperatively, taking into account the level of the intended surgery and the patient’s ASA classification.

Furthermore, Protopapa et al. [10] developed the Surgical Outcome Risk Tool (SORT) to identify patients who are at a high risk of experiencing postoperative complications and mortality. This tool assists in the strategic allocation of critical care resources. Subsequently, the parameters of the complication scoring system were enhanced using the SORT scale as a foundation, leading to improved accuracy in predicting the occurrence of postoperative complications.

Previous studies have confirmed the applicability of these scores in open gastrectomy [11–14]. However, although laparoscopic gastrectomy has been widely used in the treatment of GC, little attention has been paid to the applicability of these scoring systems to laparoscopic gastrectomy. Furthermore, surgical risk scores are constantly being modified with the advent of minimally invasive surgical techniques. Considering the difference between laparoscopic radical gastrectomy and open surgery, it is necessary to explore whether these scores are applicable to laparoscopic surgery and which score performs better. In view of this, we evaluated the applicability of POSSUM and its modified score, E-PASS and its modified score, SRS score, and SORT score in predicting postoperative mortality and morbidity after laparoscopic surgery.

## Material and methods

### IRB approval

This study was approved by the Ethical Committee of Tianjin Medical University General Hospital (ethical no. IRB2022-WZ-207).

### Patients

This retrospective study was conducted at Tianjin Medical University General Hospital from January 2016 to December 2021. The study included patients who were histopathologically diagnosed with gastric adenocarcinoma and underwent R0 resection. The exclusion criteria encompassed patients who underwent conversion to laparotomy, had incomplete data associated with the scoring systems, received preoperative radiotherapy and chemotherapy, and had multiple primary cancers. Prior to surgery, all enrolled patients underwent routine admission examination and preoperative preparations, which consisted of physical examination, laboratory tests, chest and abdomen imaging, and cardiopulmonary function evaluation. Tumor characteristics, including location, size, and lymph node metastasis, were assessed by whole abdominal contrast-enhanced CT, electronic gastroscopy, and pathologic diagnosis. Postoperative complications were graded according to the Clavien‒Dindo grading system [15] (Table 1), and patients graded II or higher were included in the study. Both morbidity and mortality from postoperative complications were defined as events occurring within 30 days of surgery.

**Table 1:** Classification of surgical complications

Grade	Definition
Grade I	Any deviation from the normal postoperative course without the need for pharmacological treatment or surgical, endoscopic, and radiological interventions
	Allowed therapeutic regimens are the following: drugs as antiemetics, antipyretics, analgetics, diuretics, electrolytes, and physiotherapy. This grade also includes wound infections opened at the bedside
Grade II	Requiring pharmacological treatment with drugs other than such allowed for grade I complications
	Blood transfusions and total parenteral nutrition are also included
Grade III	Requiring surgical, endoscopic, or radiological intervention
Grade IIIa	Intervention not under general anesthesia
Grade IIIb	Intervention under general anesthesia
Grade IV	Life-threatening complication (including CNS complications)^a^ requiring IC/ICU management
Grade IVa	Single organ dysfunction (including dialysis)
Grade IVb	Multiorgan dysfunction
Grade V	Death of a patient

^a^Brain hemorrhage, ischemic stroke, subarrachnoidal bleeding but excluding transient ischemic attacks. *CNS* Central nervous system, *IC *Intermediate care, *ICU *Intensive care unit

### POSSUM, P-POSSUM, and O-POSSUM systems

The PS variables comprised cardiac signs, chest X-ray findings, respiratory signs, systolic blood pressure, pulse rate, Glasgow Coma Index, hemoglobin level, white blood cell count, blood urea nitrogen, serum potassium, serum sodium, and electrocardiogram performance. Meanwhile, the OS variables encompassed the size of the operation, timing of the operation, blood loss, degree of peritoneal contamination, the presence of malignancy, and cumulative number of operations. The PS and OS scores were then applied to the POSSUM early complication and morbidity and mortality equations to determine the percent risk.

### E-PASS and mE-PASS system

The PRS comprised six variables, including age, the presence of significant cardiac or pulmonary disease, diabetes, performance status index [16], and ASA score. Meanwhile, the SSS consists of three variables: blood loss to body weight ratio, operative time, and type of surgical incision. The CRS is derived from both the PRS and the SSS. The PRS and SSS scores were initially calculated using the E-PASS scoring equation. Subsequently, the CRS score, representing the early postoperative morbidity and mortality rate, was calculated from the PRS and SSS scores.

### SRS system

The SRS scores are determined based on the aforementioned grading system. Subsequently, the predicted mortality was calculated by substituting the SRS score into a binary logistic equation.

### SORT system

The SORT score comprised six variables: ASA class, surgical urgency, surgical severity, surgical specialty category, the presence of cancer, and age. The risk score was calculated using patient information and subsequently incorporated into an equation to estimate the early postoperative morbidity and mortality rate [10, 17].

### Statistical analysis

A descriptive analysis was conducted on the patients included in the study, with continuous variables presented as the mean and standard deviation. Categorical variables are expressed as numbers and percentages. Demographic differences among all patients were examined. Statistical analysis for continuous data employed either Student’s *t*-test or the Mann‒Whitney *U*-test, while Pearson’s *χ*^2^ test was utilized for categorical data. A significance level of *P* < 0.05 was considered indicative of a statistically significant difference. The prognostic performance of the seven scoring systems in predicting postoperative complications, morbidity, and mortality was assessed using the area under the ROC curve (AUC). The Hosmer‒Lemeshow test was employed to evaluate the agreement between predicted mortality according to the scoring systems and the actual mortality observed in patients. *P* ≥ 0.05 was considered an indication of good predictive calibration for surgical scores. Statistical analysis was performed using SPSS 25.0.

## Results

### Clinicopathological characteristics of the patients

A total of 349 patients who underwent laparoscopic radical gastrectomy for gastric cancer (GC) were enrolled in this study. The group that experienced postoperative complications exhibited several distinguishing characteristics, including older age, lower hemoglobin count, higher bleeding volume, longer operative time, higher performance status index, and higher ASA class (Table 2). Specifically, the complication group demonstrated the highest proportion of grade 1 performance status index (5.2%) and ASA grade 3 (7.7%). Conversely, the non-complication group had the highest proportions of grade 0 (54.1%) and grade 2 (58.5%) performance status indexes. Out of the total, 42 patients (12%) experienced postoperative complications, with lung infections occurring in 10 patients (2.9%) (Table 3). Additionally, four patients (1.1%) succumbed to postoperative mortality, which comprised one case of pulmonary embolism, two cases of intra-abdominal hemorrhage, and one case of infectious shock resulting from intra-abdominal infection.

**Table 2:** Clinicopathological characteristics of the patients

	Non-complications	Complications	***P***
Age (years)	63.6 ± 9.7	72.9 ± 9.3	< 0.001
Gender			0.015
Female	106 (30.4)	9 (2.6)	
Male	201 (57.6)	33 (9.4)	
Systolic blood pressure(mmHg)	131.1 ± 13.7	135.0 ± 14.7	0.114
Hemoglobin (g/L)	120.8 ± 23.5	114.2 ± 22.9	0.046
Bleeding volume (ml)	80.6 ± 58.1	109.8 ± 84.6	0.041
Operation time (min)	248.5 ± 44.7	274.3 ± 50.1	0.001
Bleeding volume/body weight (g/kg)	1.2 ± 1.1	1.9 ± 1.8	0.015
Surgical incision (cm)	5.4 ± 1.3	6.2 ± 1.6	0.053
BMI (kg/m^2^)	22.7 ± 3.3	23.1 ± 3.2	0.405
Hospital stay (days)	14.8 ± 5.2	26.8 ± 8.9	< 0.001
Extent of resection			0.125
Proximal stomach	5 (1.4)	0 (0.0)	
Distal stomach	273 (78.2)	34 (9.7)	
Whole stomach	29 (8.3)	8 (2.4)	
TNM stage			0.053
Phase I	115 (33.0)	4 (1.1)	
Phase II	68 (19.5)	9 (2.6)	
Phase III	124 (35.5)	29 (8.3)	
Performance status index			< 0.001
Level 0	189 (54.1)	6 (1.8)	
Level 1	97 (27.8)	18 (5.2)	
Level 2	15 (4.3)	9 (2.6)	
Level 3	0 (0.0)	4 (1.0)	
Level 4	6 (1.8)	5 (1.4)	
ASA class			< 0.001
Class 1	2 (0.6)	0 (0.0)	
Class 2	204 (58.5)	12 (3.4)	
Class 3	100 (28.7)	27 (7.7)	
Class 4	1 (0.3)	3 (1.1)	
Cardiac dysfunction			0.115
None	243 (77.3)	10 (2.9)	
Mild	52 (7.2)	29 (8.3)	
Moderate	12 (3.4)	3 (0.9)	
Severe	0 (0.0)	0 (0.0)	
Pulmonary dysfunction			0.381
None	214 (61.3)	8 (2.3)	
Mild	59 (17.0)	29 (8.3)	
Moderate	29 (8.3)	4 (1.1)	
Severe	3 (0.9)	3 (0.9)	

Values in parentheses are percentage

**Table 3:** Distribution of postoperative complications

	Number of cases	Incidence
Lung infection	10	2.9%
Lower extremity deep venous thrombosis	9	2.6%
Inflammatory bowel obstruction	9	2.6%
Anastomotic leakage	9	2.6%
Pleural effusion	8	2.3%
Pancreatic leakage	7	2.0%
Cardiac insufficiency	6	1.7%
Bacteremia	5	1.4%
Urinary tract infection	4	1.1%
Duodenal stump leak	3	0.9%
Intraperitoneal hemorrhage	3	0.9%
Respiratory failure	1	0.3%

### Predictive efficacy of seven surgical scores for postoperative complications, morbidity, and mortality

**Fig. 1:**
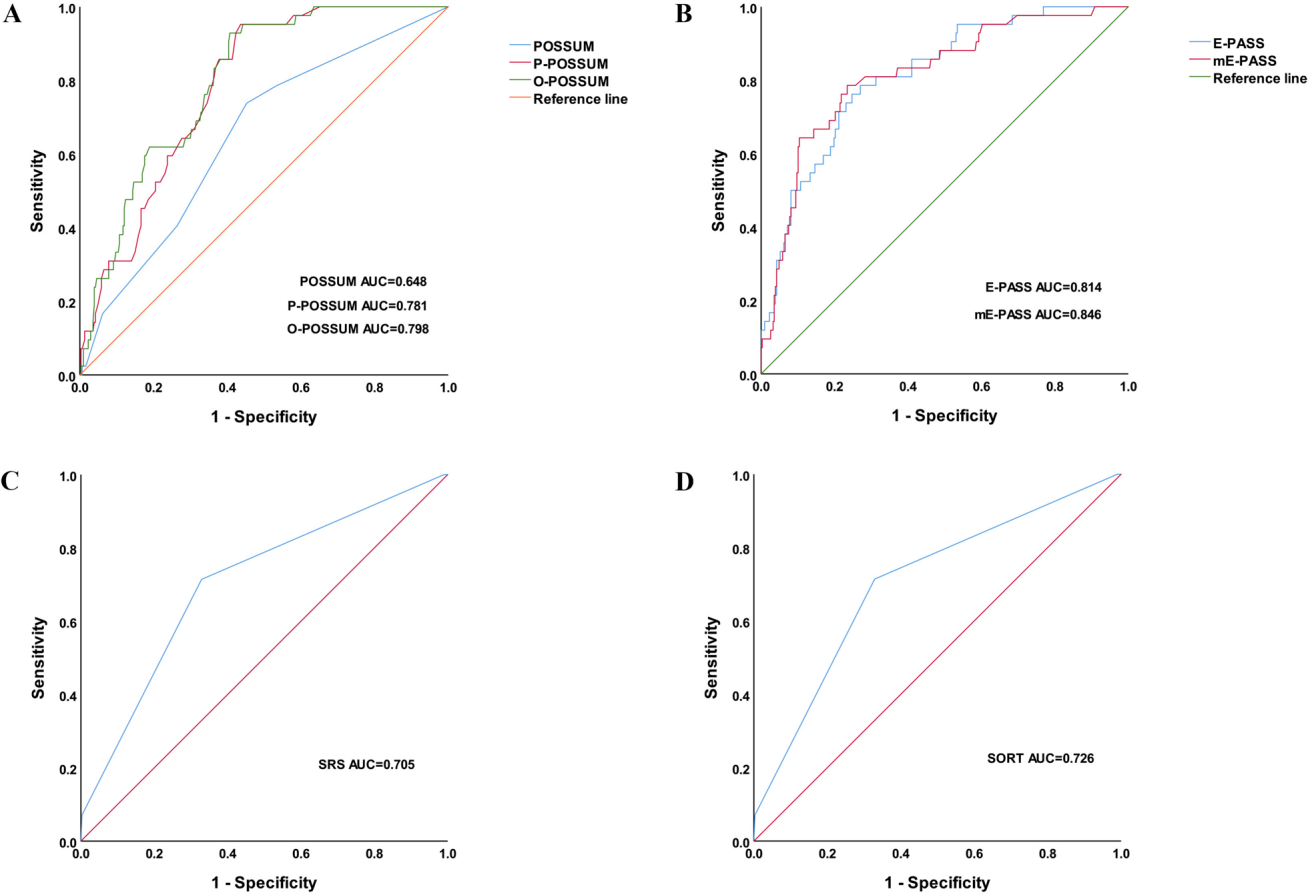
ROC curves of seven scores predicting early complication incidence

**Fig. 2:**
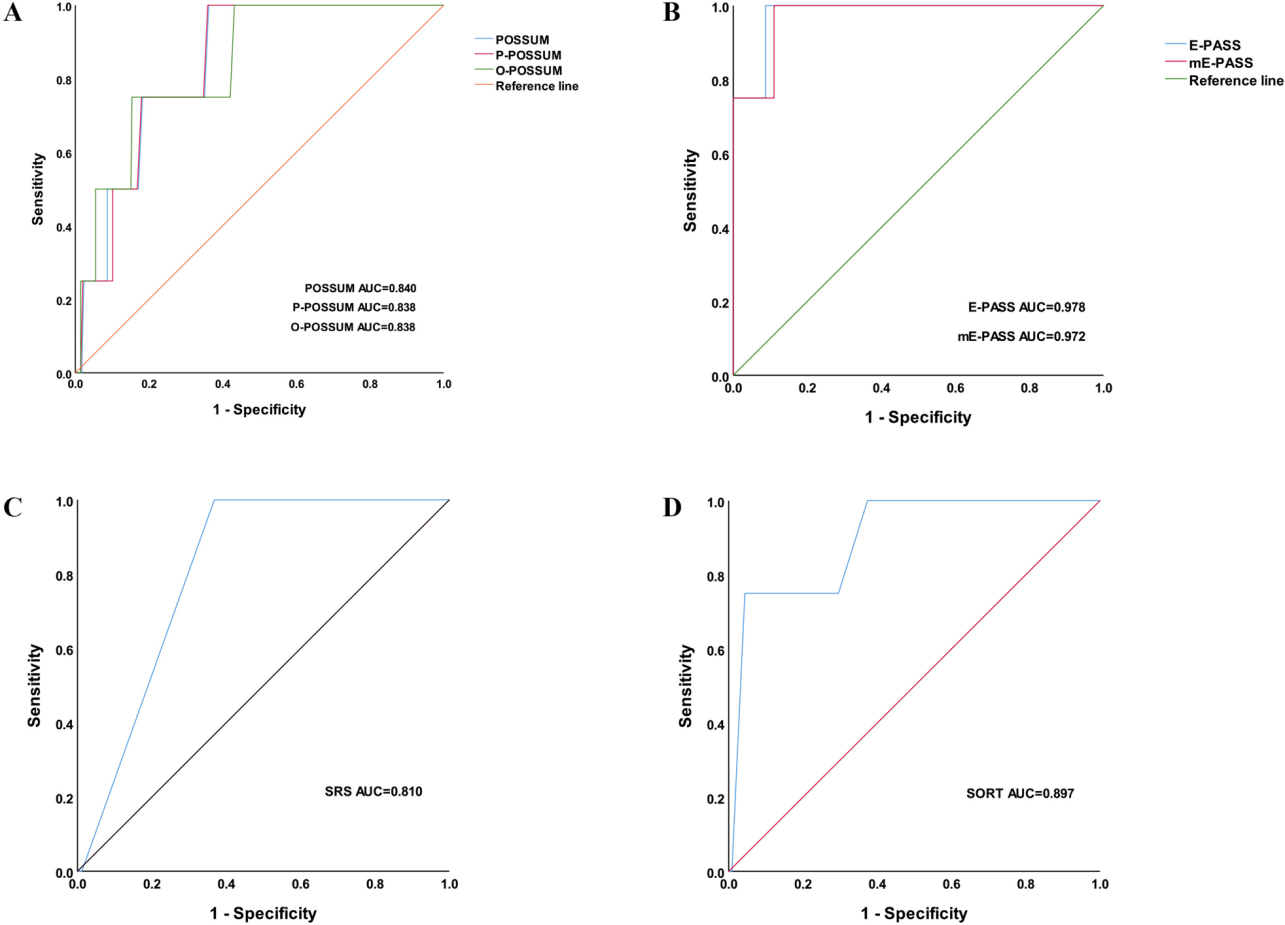
ROC curves of seven scores predicting early postoperative mortality

In this study, the discriminatory performance of seven scoring systems was evaluated using the area under the receiver operating characteristic curve (AUC). The mE-PASS scoring system demonstrated the highest AUC (0.846; 95% *CI*, 0.797–0.896; *P* < 0.001) and exhibited superior sensitivity and specificity (81% and 79%, respectively) in predicting postoperative complication morbidity (Fig. 1). Surprisingly, the POSSUM scoring system had the lowest AUC value of 0.648 (95% *CI*, 0.562–0.734;* P* = 0.002), indicating lower effectiveness in predicting complications. For postoperative mortality, the highest AUC of 0.978 (95% *CI*, 0.940–1.000; *P* = 0.001) was observed with the E-PASS score. The mE-PASS score also showed excellent discrimination with an AUC of 0.972 (95% *CI*, 0.924–1.000;* P* = 0.001), accompanied by the highest sensitivity (76%) and specificity (90%). Other scoring systems showed moderate discriminatory ability (Fig. 2). The Hosmer‒Lemeshow goodness-of-fit test indicated good agreement between the predicted and actual values for each scoring system. Detailed results of all postoperative scoring systems for predicting morbidity and mortality are shown in Tables 4 and 5. Among them, the P-POSSUM, O-POSSUM, E-PASS, mE-PASS, SRS, and SORT scoring systems were considered useful tools for predicting postoperative outcomes after laparoscopic radical gastrectomy. Notably, the mE-PASS scoring system showed the highest predictive power, while the POSSUM scoring system was only applicable for mortality prediction.

**Table 4:** Efficacy of each scoring system in predicting early complications after laparoscopic radical gastrectomy

	AUC	The optimal cutoff value (sensitivity, specificity)	*P*	Goodness-of-fit test	*P*
POSSUM	0.648	0.15 (74%; 55%)	0.002	6.077	0.193
SRS	0.705	0.26 (67%; 69%)	< 0.001	0.114	0.735
SORT	0.726	0.07 (73%; 72%)	< 0.001	0.800	0.938
P-POSSUM	0.781	0.33 (71%; 67%)	< 0.001	6.012	0.237
O-POSSUM	0.798	0.27 (76%; 66%)	< 0.001	7.236	0.512
E-PASS	0.814	0.02 (73%; 76%)	< 0.001	8.543	0.382
mE-PASS	0.846	0.50 (81%; 79%)	< 0.001	8.179	0.416

**Table 5:** Efficacy of each scoring system in predicting early mortality after laparoscopic radical gastrectomy

	AUC	The optimal cutoff value (sensitivity, specificity)	*P*	Goodness-of-fit test	*P*
SRS	0.810	0.07 (72%; 64%)	0.033	7.340	0.500
POSSUM	0.840	0.12 (75%; 80%)	0.019	1.679	0.195
P-POSSUM	0.838	0.07 (74%; 73%)	0.020	6.454	0.092
O-POSSUM	0.838	0.06 (75%; 74%)	0.020	9.069	0.337
SORT	0.897	0.16 (75%; 78%)	0.006	6.830	0.555
E-PASS	0.978	0.34 (75%; 86%)	0.001	6.697	0.521
mE-PASS	0.972	0.08 (76%; 90%)	0.001	7.442	0.492

## Discussion

The minimally invasive approach of laparoscopic gastrectomy offers advantages over open gastrectomy in terms of postoperative complications. Studies have shown that laparoscopic gastrectomy is associated with a lower incidence of complications [18–20]. The scoring systems involved in this study had been used to accurately predict postoperative morbidity and mortality in patients undergoing open surgery. Luna et al. [21] found that POSSUM and O-POSSUM overestimated postoperative mortality in open surgery, while P-POSSUM underestimated postoperative mortality. In our study, the P-POSSUM and O-POSSUM scores performed moderately for the prediction of postoperative complication morbidity in laparoscopic surgery, while the POSSUM score performed poorly. The POSSUM scoring system was initially developed and validated using data from open surgery, which may restrict its application in laparoscopic surgery due to technical and empirical factors. Additionally, there are potential variations in physiological responses and stress levels between patients undergoing laparoscopic gastrectomy and open gastrectomy. Laparoscopic surgery involves the use of pneumoperitoneum to enhance visualization, which may influence the patient’s physiological state. Our results suggest that POSSUM is not suitable for predicting postoperative complications after laparoscopic surgery. Furthermore, the predictive performance of all three scores was moderate in terms of postoperative mortality prediction, indicating their potential for evaluating the mortality rate in laparoscopic radical gastrectomy for gastric cancer. It is worth noting that the radical resections for gastric cancer in this study were conducted on an elective basis, ensuring optimal preoperative preparation and examination and allowing for the improvement of electrolyte imbalance or hypoalbuminemia in surgical patients. The similar AUC values among the scores can be attributed to these factors.

Kondo et al. [22] studied the predictive value of the E-PASS for surgical risk in very elderly patients with colorectal cancer and believed that the E-PASS was a reliable predictive score for postoperative complications. Similarly, there are few studies on the applicability of the E-PASS and mE-PASS in laparoscopic gastrectomy. In this study, E-PASS and mE-PASS showed excellent predictive effects in predicting complication incidence and mortality. Both of them could be used to evaluate the mortality and complication incidence of laparoscopic GC surgery. The results were consistent with previous reports. Different from POSSUM and its modified score, the physiological part of E-PASS and mE-PASS did not include any imaging and laboratory tests but paid more attention to the cardiopulmonary function and physical reserve of patients before surgery, which may be more suitable for features of laparoscopic radical gastrectomy. The POSSUM, SRS, and SORT scoring systems were initially developed by British scholars, whereas the E-PASS score was developed by Japanese researchers. In this study, the participants comprised a group of Chinese individuals, suggesting that the E-PASS scores, specifically designed for Asians, may be more suitable for this population. This finding provides an explanation for the superior performance of the E-PASS and mE-PASS scores, especially the mE-PASS score, which simplified the surgical variables while still showing good discriminative ability. However, it is crucial to acknowledge the influence of racial differences. Therefore, future research endeavors should encompass diverse populations to comprehensively evaluate the applicability of these scoring systems to a broader range of ethnicities.

In a prospective observational cohort study involving 202 patients undergoing various major general surgical procedures, the POSSUM, P-POSSUM, and Surgical Risk Scale (SRS) scores demonstrated comparable performance in assessing postoperative mortality and complication incidence [23]. Additionally, a retrospective study encompassing general surgery and urology revealed that the SRS, POSSUM, and P-POSSUM scores were equally effective in predicting surgical patient mortality [24]. However, there is a paucity of reports on the utilization of SRS specifically in laparoscopic radical gastrectomy; thus, the predictive capabilities of SRS in this context remain uncertain. Our study demonstrated that the SRS score displayed moderate efficacy in predicting postoperative complication incidence and mortality following laparoscopic radical gastrectomy. While SRS has the advantage of utilizing variables that can be collected preoperatively, it did not perform as prominently as the E-PASS and mE-PASS scores in predicting complication morbidity and mortality. This suggests that the variables encompassed by the SRS may be more suitable for comparative analysis across different surgical procedures, rather than for assessing patients undergoing laparoscopic radical gastrectomy.

The SORT score possesses the advantage of utilizing exclusively preoperative variables, enabling the prediction of mortality prior to surgery. The SORT score has been externally validated in patient populations undergoing liver resection and colorectal cancer surgery, establishing its credibility [25, 26]. While the Prediction of Surgical Morbidity Score (POSSUM) was superior in terms of discrimination and calibration in predicting postoperative complications, our study found that SORT exhibited moderate discrimination in predicting postoperative mortality and outperformed POSSUM in estimating postoperative complication morbidity. The SORT score incorporates three additional variables in comparison to the Surgical Risk Scale (SRS), namely, age, benign and malignant status, and surgical specialty, and its performance in this study surpasses that of the latter.

In the study, there are several limitations that should be acknowledged. Firstly, it is important to note that this study was a single-center retrospective study. Secondly, considering the potential impact of racial differences on the findings, it is crucial to exercise caution in generalizing the results. Additionally, it is worth mentioning that the number of deaths in the study was limited to only four cases. To assess the extent to which the surgical system scores described earlier can be applied to a broader population, future studies should aim to include a larger and more diverse study population.

## Conclusion

The P-POSSUM, O-POSSUM, E-PASS, mE-PASS, SRS, and SORT scoring systems are available tools for predicting postoperative outcomes of laparoscopic radical gastrectomy. The mE-PASS scoring system showed the best predictive power and could be a powerful predictive tool for predicting postoperative outcomes of laparoscopic radical gastrectomy. However, the POSSUM scoring system could only be applicable to the prediction of postoperative mortality.

## Data Availability

All data, materials, and operation videos used during the study are available from the corresponding author by request.
